# Downregulation of *Aedes aegypti* chromodomain helicase DNA binding protein 7/Kismet by *Wolbachia* and its effect on dengue virus replication

**DOI:** 10.1038/srep36850

**Published:** 2016-11-09

**Authors:** Sultan Asad, Sonja Hall-Mendelin, Sassan Asgari

**Affiliations:** 1 Australian Infectious Disease Research Centre, School of Biological Sciences, The University of Queensland, Brisbane QLD 4072 Australia; 2 Virology, Public and Environmental Health, Forensic and Scientific Services, Department of Health, Queensland Government, Brisbane, QLD, Australia

## Abstract

Dengue virus (DENV) is a mosquito-transmitted virus imposing a significant burden on human health around the world. Since current control strategies are not sufficient, there is an urgent need to find alternative methods to control DENV transmission. It has been demonstrated that introduction of *Wolbachia pipientis* in *Aedes aegypti* mosquitoes can impede DENV transmission with the mechanism(s) not fully understood. Recently, a number of studies have found the involvement of chromodomain DNA binding helicases in case of Human Immunodeficiency virus (HIV) and Influenza A virus infection. In this study, we have identified three chromodomain helicase DNA binding protein (*CHD*) genes in *Ae. aegypti* and looked at their response in the case of *Wolbachia* and DENV infections. Foremost amongst them we have found that *AeCHD7*/*Kismet* is significantly downregulated in the presence of *Wolbachia* infection only in female mosquitoes. Furthermore, *AeCHD7* levels showed significant increase during DENV infection, and *AeCHD7* depletion led to severe reduction in the replication of DENV. Our data have identified *AeCHD7* as a novel *Ae. aegypti* host factor that is important for DENV replication, and *Wolbachia* downregulates it, which may contribute towards the mechanism(s) of limiting DENV replication.

Among arboviruses, dengue virus (DENV) is one of the most important flaviviruses having the potential to affect two-thirds of the world’s population^1^,^2^. DENV is primarily transmitted to humans through the bit of mosquito vector *Aedes aegypti*, leading to dengue infection and potentially dengue haemorrhagic fever^3^,^4^,^5^. Lack of availability of an effective vaccine and proper medical care has narrowed DENV management strategies to vector control. One of the strategies used to overcome DENV vector *Ae. aegypti* is through the application of pesticides, but due to their severe consequences on the environment and the emergence of resistance to pesticides, their potential application seems bleak in the near future^6^. Therefore, new strategies for vector control are urgently needed. One of the novel options is the use of an endosymbiotic bacterium *Wolbachia* which has recently been demonstrated to limit DENV, West Nile virus (WNV), and Zika virus (ZIKV) replication in *Ae. aegypti*^7^,^8^,^9^,^10^.

*Wolbachia pipientis* is an alphaproteobacterium that naturally infects almost 40–60% of insect species^11^,^12^. This bacterium is maternally transmitted and is usually associated with manipulations of host reproduction, such as feminization^13^ and male killing^14^, to promote successful colonization of its host species. *Wolbachia* naturally infects several mosquito species, including *Aedes albopictus* and *Culex pipiens*^15^. However, there is no natural *Wolbachia* infection in the case of *Ae. aegypti*, which is the most notorious vector for several arboviruses. In order to exploit *Wolbachia*’s potential to limit arbovirus transmission, three strains of *Wolbachia*, *w*AlbB (from *Ae. albopictus*)^16^, *w*Mel (from *Drosophila melanogast*er)^17^ and *w*MelPop-CLA (from *D. melanogaster*)^18^ have been successfully transinfected into *Ae. aegypti*. Among these three strains, *w*Mel and *w*MelPop-CLA are the most promising ones for virus blocking^7^,^8^,^9^,^10^,^19^,^20^. However, the exact mechanism(s) by which *Wolbachia* blocks viral replication in *Ae. aegypti* mosquitoes is still elusive. Few studies that have looked into the transcriptional changes in *Ae. aegypti* mosquitoes upon *Wolbachia* infection have found increased redox and mitochondrial activity along with differential serine protease activity^21^,^22^,^23^. However, very little is known about the role of chromatin remodelers in the case of DENV-*Aedes-Wolbachia* molecular interactions.

Chromodomain helicase DNA binding proteins (*CHD*) represent a class of ATP-dependent chromatin remodelling enzymes that contribute towards invoking changes in the interaction between DNA and nucleosomes^24^, influencing a wide array of cellular processes such as replication, transcription, recombination, repair and development^25^. Members of the CHD family have been found to be involved in replication of Human Immunodeficiency virus (HIV) and Influenza A virus^26^,^27^. All the CHD protein family members have a pair of chromodomains at their N-terminus along with one sucrose non-fermenting (SNF2) domain in the centre^25^. In humans, the CHD family has nine members. These are further classified, on the basis of additional motif features, into three subfamilies: CHD1-2 (class I), CHD3-5 (Class II) and CHD 6-9 (class III)^25^,^28^. In *D. melanogaster*, there are three well characterized CHD members named CHD1^29^, Mi2^30^ and Kismet/CHD7^31^. CHD1 is essential for the fecundity of both males and females and is indirectly involved in transcriptional elongation^32^, whilst Mi2 actively participates in transcriptional repression and is vital for expression of heat shock proteins^33^,^34^. *Drosophila* Kismet, that is a homolog of human CHD7, mediates transcriptional elongation^35^. Apart from characterization of the CHD family members’ role in development and chromatin modification, very little is known about their potential role in host-pathogen interactions.

In this study, we have identified functional homologs of the CHD family members in *Ae. aegypti* and looked at the effect of *Wolbachia* infection on their expression. There was significant reduction in the expression of all *CHD* family members in the presence of *Wolbachia*. Furthermore, we found that *AeCHD7* is highly induced during DENV infection in *Ae. aegypti* mosquitoes. A silencing assay demonstrated that *AeCHD7* is required for the efficient replication and virion production of DENV. This study will help to understand the role of *AeCHD7* in DENV-*Aedes-Wolbachia* interactions.

## Results

### Screening of the CHD family genes during *Wolbachia* infection

**Figure 1:**
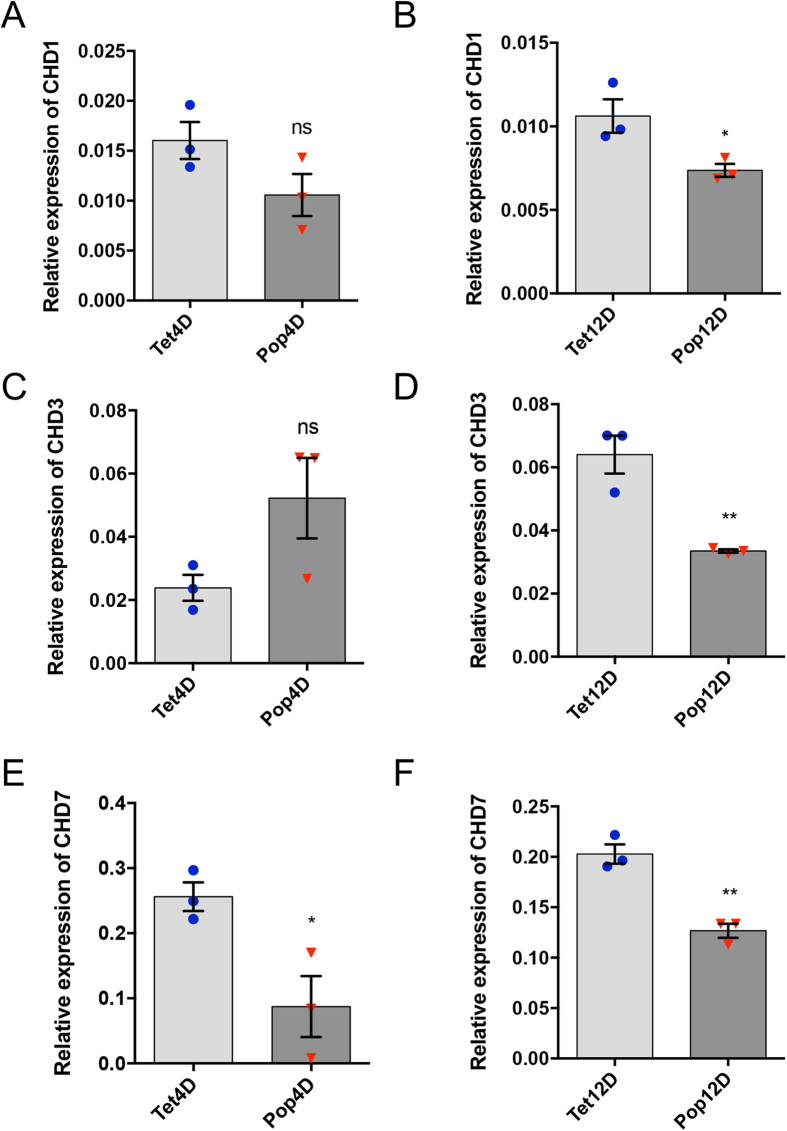
Relative expression of *AeCHD* genes in uninfected and *Wolbachia-*infected *Ae. aegypti* mosquitoes. RT-qPCR based quantification of (**A**,**B**) *AeCHD1*, (**C**,**D**) *AeCHD3*, and (**E**,**F**) *AeCHD7* genes in both *Wolbachia*-infected (Pop) and uninfected (Tet) 4-day-old and 12-day-old female mosquitoes, respectively. Error bars represent standard error of mean (SEM) from three biological replicates (*p < 0.05; **p < 0.01).

Three *CHD* genes were identified in the *Ae. aegypti* genome using Vectorbase^36^. Blastp was run to identify their homologs in *D. melanogaster* and *Culex quinquefasciatus*, and these were determined as *AeCHD1* (AAEL004716) having 58% identity with *D. melanogaster* CHD1 protein (NP_477197.1), *AeCHD3* (AAEL013136) that showed 70% identity with *D. melanogaster* CHD3 protein (AAD17276.1) and *AeCHD7* (AAEL002230) showing 58% identity with *D. melanogaster* Kismet/CHD7 protein (NP_001245820.1). qPCR primers were designed for all the three *AeCHD* family members to experimentally validate their expression in *Ae. aegypti* mosquitoes by RT-qPCR, and the effect of *Wolbachia* (*w*MelPop) infection on their expression level. For this, we selected two age groups of *Ae. aegypti* mosquitoes, 4-day- and 12-day-old. While expression of all the three *AeCHD* genes was confirmed in the mosquitoes, they were all mostly downregulated in *Wolbachia*-infected mosquitoes (Fig. 1A–F), except for *AeCHD3*, which was found to be non-significantly upregulated in 4-day-old *Wolbachia*-infected mosquitoes (Fig. 1C). However, *AeCHD7* showed the highest change of 2.9-fold downregulation in 4-day-old *Ae. aegypti* female mosquitoes (Fig. 1E), which led us to further characterise the gene.

### *AeCHD7* is ubiquitously expressed in all mosquito tissues

**Figure 2:**
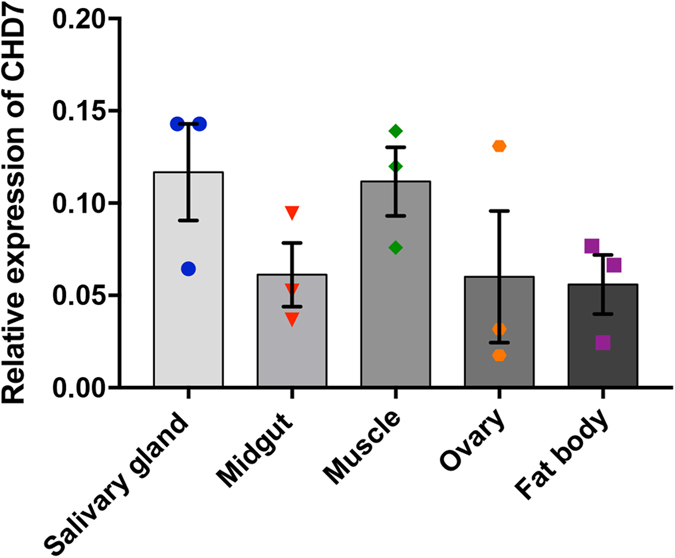
Tissue-specific expression of *AeCHD7* in *Ae. aegypti* mosquitoes. RT-qPCR results of *AeCHD7* transcript levels in the salivary gland, midgut, muscles, ovaries and fat body of 3-day-old tetracycline treated female mosquitoes. Error bars represent SEM of the mean in three biological replicates.

In order to determine the relative abundance of *AeCHD7* across different tissues, the salivary gland, midgut, muscle, ovary and fat body were isolated from 3-day-old female *Ae. aegypti* mosquitoes. Following RT-qPCR detection of *AeCHD7* mRNA transcripts, it was found that *AeCHD7* is ubiquitously expressed in all tissues with the highest expression level in the salivary gland, which was 2.1-fold higher than its expression level in the fat body which showed the lowest relative abundance of *AeCHD7* transcripts (Fig. 2). These results are consistent with the previous findings which showed that *AeCHD7* is expressed in all human tissues^37^.

### Specific *Wolbachia*-mediated downregulation of *AeCHD7* in female *Ae. aegypti*

**Figure 3:**
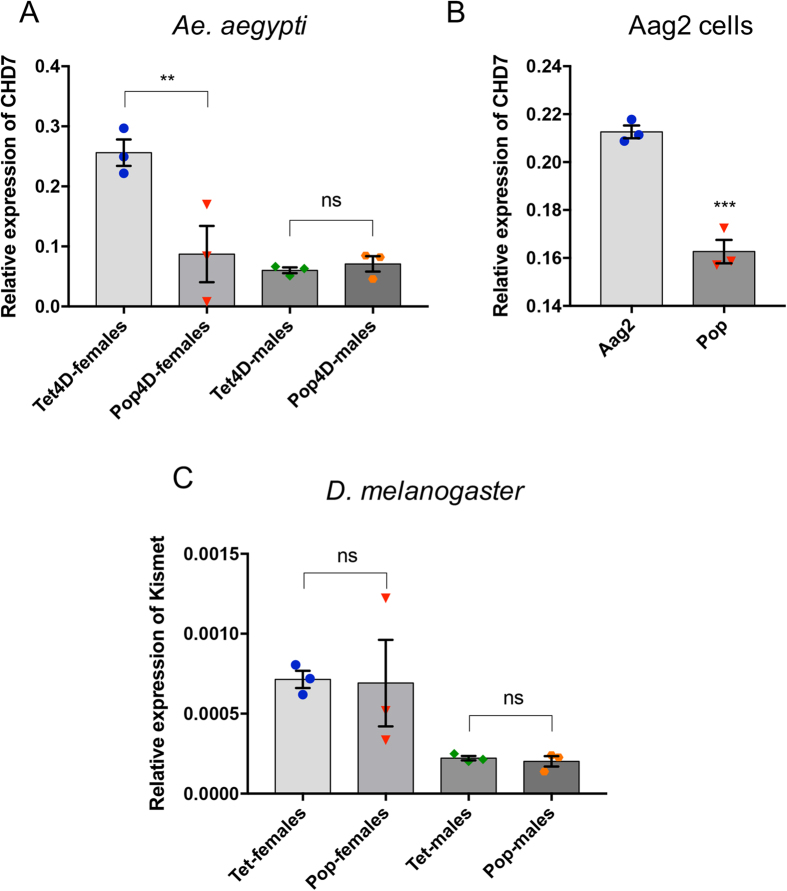
Modulation of *AeCHD7/Kismet* by *Wolbachia* infection in male and female mosquitoes and flies, and mosquito cell lines. (**A**) RT-qPCR analysis of *AeCHD7* transcript levels in 4-day-old female and male mosquitoes, both uninfected (Tet) and infected with *Wolbachia* (Pop). (**B**) Relative expression of *AeCHD7* in Aag2 and Aag2 cells infected with *w*MelPop-CLA (Pop). (**C**) Relative expression of the *D. melanogaster Kismet* gene in uninfected (Tet) and *Wolbachia-*infected (Pop) flies. Error bars represent SEM from three biological replicates (**p < 0.01; ***p < 0.001; ns, not significant).

To find out whether *Wolbachia*-mediated downregulation of *AeCHD7* is gender specific, we evaluated the transcript levels of *AeCHD7* in 4-day-old female and male *Ae. aegypti* mosquitoes with and without *Wolbachia* infection. RT-qPCR results showed that *Wolbachia* downregulates *AeCHD7* only in female mosquitoes and not in their male counterparts (Fig. 3A). This is interesting in the sense that *Wolbachia* has a gender specific effect on gene expression in the mosquitoes. To examine if the effect can consistently be seen in cell lines as well, we cross-validated the *AeCHD7* mRNA expression levels in *Ae. aegypti* cell lines, Aag2 and Aag2 infected with *w*MelPop-CLA (Pop) and found a similar trend of *AeCHD7* transcript downregulation in *Wolbachia*-infected cells (Fig. 3B).

Furthermore, to evaluate if *Wolbachia* has a similar effect on the *CHD7* gene in its natural host *D. melanogaster*, four 7-day-old male and female flies infected with *Wolbachia* (*w*MelPop strain) were examined for the relative expression of *CHD7*. RT-qPCR results confirmed that there was no significant change in the level of *CHD7* mRNA in both male and female *D. melanogaster* flies infected with *Wolbachia* (Fig. 3C).

### *AeCHD7* is upregulated upon DENV infection

**Figure 4:**
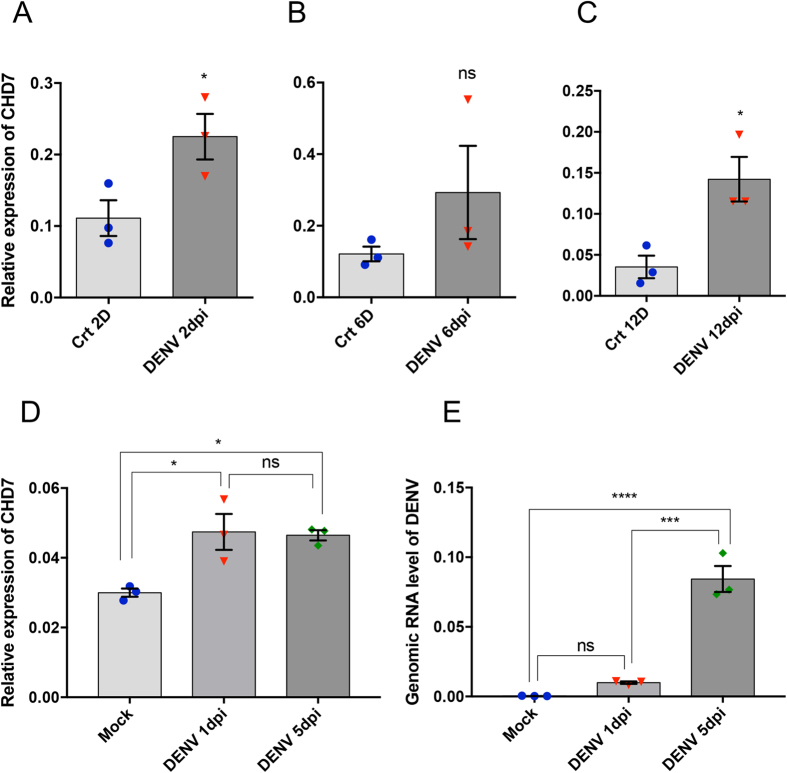
Expression pattern of *AeCHD7* in DENV infected female *Ae. aegypti.* (**A**–**C**) RT-qPCR quantification of *AeCHD7* transcript levels at 2, 6 and 12 days post DENV infection of female *Ae. aegypti* mosquitoes. (**D**) Relative transcript levels of *AeCHD7* in Aa20 cells infected with 1 MOI of DENV2 analysed at 1 and 5 dpi. (**E**) RT-qPCR quantification of DENV2 genomic RNA in samples used in (**D**) confirming virus infection and replication. Error bars show SEM from three biological replicates (*p < 0.05; ***p < 0.001; ****p < 0.0001; ns, not significant).

Considering the virus blocking effect of *Wolbachia* in *Ae. aegypti* mosquitoes, we examined the transcript levels of *Ae*CHD7 in the context of mosquito-DENV interaction. For this, the transcript levels of *AeCHD7* in DENV-injected mosquitoes at three different time points of 2, 6 and 12 days post-infection (dpi) were analysed. To have a more consistent and high success rate of DENV infection, mosquitoes were injected rather than orally fed with the virus. The results revealed that there was an increase in the *AeCHD7* transcript levels upon DENV infection at all the time points (Fig. 4A–C); however, the upregulation was only significant at 2 and 12 dpi, which was 2-fold (Fig. 4A) and 4-fold higher than that in uninfected mosquitoes (Fig. 4C), respectively. The aforementioned findings were further confirmed in the *Ae. aegypti* cell line, Aa20. Cells were infected with DENV2 at 0.1 multiplicity of infection (MOI) and were harvested at two different time points that were 1 and 5 dpi. RT-qPCR analysis showed significant increase in *AeCHD7* transcript levels in the case of DENV infection at both 1 and 5 dpi (Fig. 4D). Infection was confirmed by relative quantification of DENV genomic RNA levels, which showed gradual increase in DENV genomic RNA over time (Fig. 4E).

### *AeCHD7* is required for efficient DENV replication

**Figure 5:**
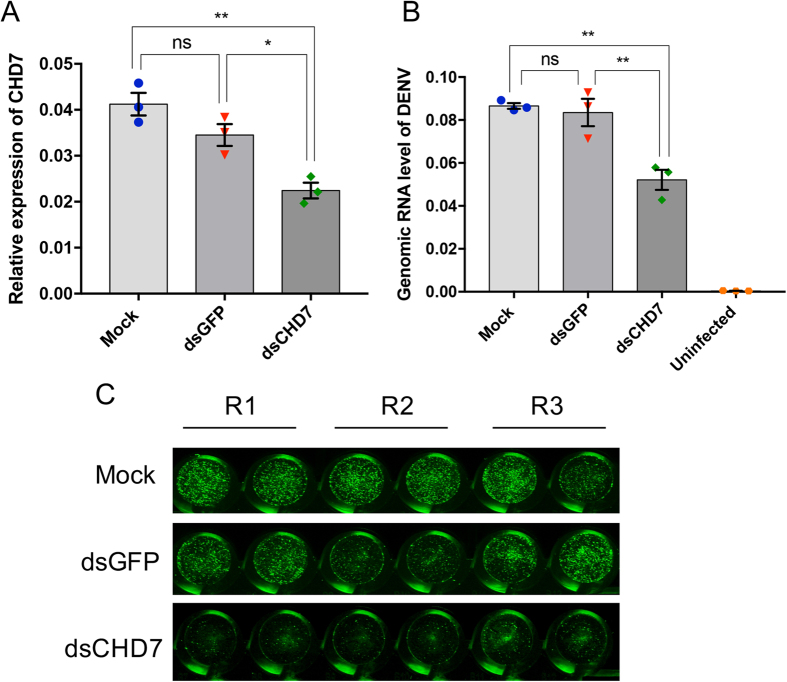
Depletion of *AeCHD7* impairs DENV replication both at the genomic and the virion levels. (**A**) RT-qPCR analysis of Aa20 cells transfected with either no RNA (Mock) or with dsRNA against GFP as a control or with dsRNA against *AeCHD7*. *RPS17* was used to normalize the qPCR data. Error bars show SEM from three biological replicates (*p < 0.05; **p < 0.01). (**B**) RT-qPCR analysis of Aa20 cells treated as in (**A**) followed by DENV infection at 1 MOI using DENV-specific primers to quantify viral genomic RNA. Error bars show SEM from three biological replicates (**p < 0.01). (**C**) Viral plaque visualization by *in vitro* cell plaque assay conducted on the supernatant media from cells treated as in (**A**,**B**).

The upregulation of *AeCHD7* in DENV-infected cells suggested that the gene could be beneficial for the virus. To investigate whether *AeCHD7* is required for efficient DENV replication, we knocked down *AeCHD7* transcripts in Aa20 cells and challenged these cells with DENV at 1 MOI for 72 h. The effect of *CHD7* knockdown on DENV was evaluated both at the genomic and the virion levels using RT-qPCR and plaque assay. RT-qPCR results confirmed ~50% decrease in *AeCHD7* mRNA level (Fig. 5A), which led to 2-fold reduction in DENV genomic RNA (Fig. 5B). RT-qPCR results were further validated with plaque assay, which confirmed reduction in DENV virion production in *AeCHD7* knocked down cells as compared with dsGFP or mock-transfected Aa20 cells (Fig. 5C). These results indicate that *AeCHD7* is a host factor that is used by DENV to facilitate its replication in *Ae. aegypti* female mosquitoes, and *Wolbachia* downregulates *AeCHD7* as shown above, which may contribute to restricting DENV replication.

## Discussion

There is accumulating experimental evidence showing the effectiveness of *Wolbachia* in suppressing the replication of several flaviviruses, including DENV, ZIKV, WNV, and the alphavirus chikungunya virus (CHIKV) in both mosquitoes and mosquitoes-derived cell lines^8^,^10^,^38^. Perhaps the most well studied is the case of DENV replication that is severely compromised in the presence of *Wolbachia*^39^,^40^,^41^. However, the exact mechanism(s) of how *Wolbachia* imparts this antiviral effect is not yet fully understood. In this study, we provide experimental evidence that chromodomain DNA binding helicase 7 (*AeCHD7*) is an *Ae. aegypti* host factor that is exploited by DENV to facilitate its replication, and its downregulation by *Wolbachia* may contribute to limit DENV replication.

*Wolbachia* is an endosymbiotic bacterium infecting 40–60% of insect species naturally^11^ by manipulating host reproduction^12^. Despite fitness costs, *Wolbachia* may benefit its host by blocking a variety of RNA viruses^15^,^42^. However, *Wolbachia* has not been found naturally infecting the most notorious vector *Ae. aegypti*, that is responsible for transmitting multiple viral diseases^18^. McMeniman *et al.* transinfected different strains of *Wolbachia* into *Ae. aegypti* mosquitoes^43^ and found that they successfully inhibited replication of DENV and CHIKV^38^. Further studies also demonstrated *Wolbachia*’s ability to block WNV and ZIKV in the mosquito^8^,^9^,^10^. *Wolbachia’s* potential to be used as an invaluable tool for disease control and prevention represents an increasingly promising approach to limit several mosquito-borne viral diseases, and it is fascinating to explore the exact mechanism(s) that induce the antiviral effect. Apart from one study shedding light on the effect of *Wolbachia* infection on the global DNA methylation pattern in mosquitoes^44^, there lies a huge grey area of the role of chromatin remodelers, in *Wolbachia*-host interactions and possibly the *Wolbachia*-mediated antiviral effect.

CHD proteins represent a class of proteins that belong to SNF2 superfamily of ATP-dependent chromatin modifiers. In mammals, there are 1–9 CHD proteins; however, in *D. melanogaster*, there are only three CHD proteins named CHD1, Mi2 and Kismet^25^. Members of the CHD family are involved in conducting a wide array of functions, including ATPase activity to maintain chromosome structure and regulation of heterochromatic elements^29^, nucleosome mobilization^45^, transcriptional regulation and elongation^46^,^47^,^48^,^49^, and development and differentiation^31^. Despite extensive characterization of CHD family proteins, their role in shaping host-pathogen interactions has not been much investigated. Yet, there are few reports supporting the involvement of CHD1 in the case of influenza A virus, and both CHD1 and CHD2 in the case of HIV as positive regulators^26^,^27^. Furthermore, RNAi screen carried out in *D. melanogaster* S2 cells identified the involvement of CHD7/Kismet in antimicrobial humoral response^50^. The aforementioned facts led us to investigate the possible role of the CHD family in *Wolbachia*-*Aedes*-DENV interactions. Data mining in VectorBase resulted in the identification of three potential AeCHD proteins encoded in the *Ae. aegypti* genome. Protein blast results identified them as AeCHD1, AeCHD3/Mi2 and AeCHD7/Kismet. In order to find out whether *Wolbachia* regulates the *AeCHD* genes during infection, RT-qPCR was performed to examine the transcript levels of all the three *AeCHDs* with and without *Wolbachia* infection in whole mosquitoes. Our results showed that there was a uniform trend of downregulation of the transcript levels of *AeCHD* genes in *Wolbachia*-infected mosquitoes (Fig. 1A–F), except those of *AeCHD3* in 4-day-old *Wolbachia-*infected female mosquitoes, which showed a non-significant upregulation (Fig. 1C). The reduction in the *CHD* genes was more pronounced in 12-day-old mosquitoes, which could be due to increases in the *Wolbachia* load as the mosquitoes ages. In particular, the *w*MelPop strain is a virulent strain that may cause tissue damage and sickness. The reductions in the *AeCHD* genes at this late stage may not be of benefit in affecting DENV replication. However, *AeCHD7* showed the highest fold change reduction (Fig. 1E,F) in both 4- and 12-day-old *Wolbachia*-infected mosquitoes, which prompted us to further investigate this gene in the context of *Wolbachia*-*Aedes*-DENV interactions.

AeCHD7/Kismet belongs to subfamily III of the CHD proteins that comprises CHD5-9 proteins. This subfamily is defined by the presence of two chromodomains at the N-terminus, one SNF2-like ATPase domain located in the central region of the protein structure and a Brahma and Kismet (BRK) domain at the C-terminus^25^. To find out whether the *Ae. aegypti* homolog fulfils this particular protein signature, NCBI conserved domain finder was used to detect the conserved domains^51^. Results confirmed the presence of all the domains characteristic of CHD7/Kismet proteins (Figure S1). Furthermore, in order to check the conservation of CHD7 across species, CHD7/Kismet amino acids were retrieved from Uniprot (Figure S2A) and subjected to maximum likelihood phylogenetic tree construction. Phylogenetic results showed that *Ae. aegypti* CHD7 is closely related to that of *Cx. quinquefasciatus* but not to that of *D. melanogaster* or *Homo sapiens* (Figure S2B). To find out the tissue-specific expression of *AeCHD7*, RT-qPCR was performed, which revealed that it is ubiquitously expressed across all main mosquito tissues (Fig. 2), which is consistent with the findings in humans^37^.

In this study we found that *AeCHD7* was significantly downregulated in *Wolbachia-*infected mosquitoes. To further investigate whether this *Wolbachia*-mediated downregulation of AeCHD7 in female *Ae. aegypti* is gender specific and can also be seen in its natural host *D. melanogaster*, RT-qPCR was employed. Interestingly, we found that in the presence of *Wolbachia AeCHD7* was specifically downregulated in female *Ae. aegypti* only (Fig. 3A), and there was no change in *CHD7/Kismet* transcript levels in both female and male *D. melanogaster* with and without *Wolbachia* (Fig. 3C). This difference in *Wolbachia*-mediated regulation of CHD7/Kismet in *Ae. aegypti* and *D. melanogaster* may be due to the fact that *Wolbachia* is a natural symbiont in *D. melanogaster* with a long association, while it has been recently transinfected into *Ae. aegypti*^52^. In regards to the mechanism by which *Wolbachia* infection may affect expression of *AeCHD7*, one can only speculate at this stage as there is very little information in regards to how *Wolbachia* manipulates its host at the molecular level. Only very recently, some molecular data have become available showing that *Wolbachia* infection leads to changes in the transcriptome or small RNA profiles of infected mosquitoes^23^,^53^. Regulation of host gene expression could be due to components secreted from the endosymbiont, including small non-coding RNAs^54^, or host response to accommodating the endosymbiont, in particular in new associations. However, how *Wolbachia* infection leads to these changes in the host remains to be investigated.

Viruses are the master manipulators of their host environment for their own benefit. Recently, it has been reported that CHD1 and CHD2 proteins play a pivotal role in the replication of influenza and HIV viruses^26^,^27^. Both viruses replicate inside the nucleus. Interestingly, it has further been demonstrated that CHD1 interacts with RNA polymerase II to facilitate the replication of influenza A virus^27^. Little is known about the role of CHD7/Kismet in the context of virus infection. However, the presence of conserved chromodomains and a SNF2 domain makes it highly likely that all the CHDs share similar functions^25^. We were intrigued to find what happens to AeCHD7 during DENV infection. RT-qPCR performed in DENV-infected mosquitoes at different time points suggested a continuous trend of upregulation during DENV infection (Fig. 4A–C), which points to the fact that it might play an important role in DENV replication. To investigate the role of AeCHD7 in DENV replication further, AeCHD7 knockdown study was carried out, which revealed that AeCHD7 is vital for DENV replication and virion production (Fig. 5A–C). Very few studies that have been carried out on the involvement of CHDs in virus replication have predominantly focused on viruses that replicate inside the nucleus. Therefore, the role of CHDs in the replication of viruses that multiply in the cytoplasm (such as DENV) is not known. However, it has been demonstrated that DENV capsid^55^,^56^ and NS5 proteins go inside the nucleus^57^,^58^ with NS5 known to be involved in disrupting nucleosome formation^59^. Therefore, these viral proteins may play a role in modulating AeCHD7 expression during virus infection. While we have found the involvement of AeCHD7 in mosquito-DENV interaction, the exact mechanism(s) that govern the interaction need further investigation.

In summary, we have demonstrated that AeCHD7 facilitates DENV replication, and *Wolbachia*-mediated downregulation of AeCHD7 in female *Ae. aegypti* may contribute to restriction of DENV replication. However, this mechanism is highly specific to female *Ae. aegypti* mosquitoes and does not appear to be a universal mechanism which *Wolbachia* employs across different hosts to block viral replication.

## Materials and Methods

### Mosquitoes and flies

For *Wolbachia* studies, mosquitoes had been previously generated by McMeniman *et al.*^43^ by transinfecting *w*MelPop-CLA strain of *Wolbachia* (Pop) into *Ae. aegypti* embryos, and uninfected mosquitoes were obtained through tetracycline (Tet) treatment of the infected mosquitoes^43^. *w*^*1118*^ fly line stably infected with *w*MelPop-CLA and the tetracycline cured line were generated by Min *et al.*^60^ and kindly provided by Dr Karyn Johnson from the University of Queensland.

For DENV infection studies, *Ae. aegypti* eggs were collected in Townsville in August 2015 and reared in insectary at Public Health Virology FSS. Five-day-old *Ae. aegypti* (F3) were used for the experiments. DENV2 NGC strain obtained from Prof. Roy Hall’s Lab (University of Queensland, School of Chemistry & Molecular Biosciences, Brisbane, Australia), was diluted in Opti-MEM (GIBCO Life Technologies, Grans Island, NY) supplemented with 3% foetal bovine serum (FBS, In Vitro Technologies, Australian origin) and intrathoracically injected (200 μl) in 5-day-old mosquitoes at 10^5.8^/mL (10^2.1^ per dose). Mosquitoes were placed into netted 900 mL containers at 28 °C with light:dark (L:D) 12:12 hours cycle and at high humidity. Mosquitoes were offered 15% honey water *ad libitum*. Mosquitoes were collected at 2, 6 and 12 dpi for downstream applications.

### Cell cultures

*Ae. aegypti* Aag2 cell line and Aag2 cells infected with *w*MelPop-CLA, previously described by^61^, were maintained in 1:1 Mitsuhashi-Maramorosch and Schneider’s insect medium (Invitrogen) supplemented with 5–10% FBS (Bovogen Biologicals, French origin), while Aa20 cells were maintained in L15 medium (Invitrogen) supplemented with 10% tryptose phosphate broth (TPB) and 5% FBS. All mosquito cell lines were kept at 28 °C and passaged every 3–4 days.

Vero cells were maintained in OptiMEM medium supplemented with 2% FBS and were kept at 37 °C in the presence of 5% CO_2_.

### RT-PCR and qPCR analyses

Total RNA was extracted from mosquitoes (1–5 mosquitoes per biological replicate) or flies (1–5 flies per biological replicate) using Qiazol (Qiagen) and then treated with Turbo DNase (Ambion) according to the manufacturers’ instructions. 750–1000 ng of total RNA was then used to make the 1^st^ strand cDNA using Superscript III (Invitrogen) with either oligo-dT primer for cellular transcripts or with DENV-qR primer in order to amplify the DENV genomic RNA.

For qPCR, cDNA produced as above was diluted in 1:5 ratios with nuclease free water. 2 μl of the diluted cDNA was used for downstream qPCR reaction. Both forward and reverse gene-specific primers were used to amplify the target genes (primer sequences in Table S1), using QuantiFast SYBR Green (Qiagen) in a Rotorgene qPCR machine (Qiagen). For *Ae. aegypti* samples, *RPS17* transcript levels were used for the normalization of RNA templates, while *RPL32* was used for normalization of *D. melanogaster* samples. Each qPCR reaction was performed in duplicates with at least three biological replicates. All qPCR data were normalized with Qiagen analysis templates and were further analysed by Prism 7.0. Unpaired t-test was used to determine statistical significance between two individual groups while one-way ANOVA with Tukey’s post-hoc test was performed to find statistical significance between more than two groups of data.

### RNAi-mediated gene silencing

In order to knockdown the *AeCHD7* gene for functional analysis in DENV life cycle, primers were designed to amplify a 586 bp product from the *AeCHD7* gene with the addition of the T7 promoter sequences at both ends (Table S1). MEGAscript T7 Transcription kit was then used according to the manufacturer’s instructions in order to synthesize dsRNA targeting the *AeCHD7* transcripts. A similar approach was followed to synthesize dsRNA against *GFP* RNA. For knockdown experiments, Aa20 cells were double transfected with 2–5 μg of dsRNA per well against the target gene. dsGFP RNA was used as non-specific control.

### Virus infection and plaque assay

For virus inoculation experiments, *Ae. aegypti* Aa20 were seeded at the density of 3 × 10^5^ cells per well in 12-well plates. Cells were first double transfected with dsRNA against the target gene or *GFP* control and after 6 h cells were infected with DENV2 (New Guinea strain) at a multiplicity of infection (MOI) of 1. Media were collected 72 h post-infection for plaque assay.

To perform plaque assay, Vero cells were seeded in a 96-well plate and were allowed to form monolayers. Virus containing media from the experiments were serially diluted into 10^0^, 10^1^, 10^2^, 10^3^ dilutions and added to Vero cells in duplicates. Cells were incubated with virus at room temperature with continuous shaking on shaker for 1 h and then incubated at 37 °C for one additional hour. After 2 h of incubation, media were aspirated and an overlay was added to the cells which comprised of 1.5% carboxymethyl cellulose (CMC) and 2.5% FBS in Opti-MEM medium (Sigma). Cells were then incubated for 72 h at 37 °C and 5% CO_2_ and fixed with 80% ice-cold acetone in 1X PBS for 20 min at −20 °C. Plates were then air dried overnight and blocked with 5% skimmed milk in 1 × PBST at 37 °C for 30 min. Cells were then incubated with the primary antibody against DENV2-Envelope (human) in 1:1000 dilution in 0.1% skimmed milk in 1 × PBST for 2 h at 37 °C as described previously^62^. Plates were washed 3 times with 1 × PBST and incubated with the secondary antibody (IRDye 800 CW goat anti-human LICOR) for 1 h at 37 °C. Plates were washed and dried as above and were dried and scanned on the Odyssey imager (LI-COR Biosciences) at 41 μM resolution.

## Additional Information

**How to cite this article**: Asad, S. *et al.* Downregulation of *Aedes aegypti* chromodomain helicase DNA binding protein 7/Kismet by *Wolbachia* and its effect on dengue virus replication. *Sci. Rep.* **6**, 36850; doi: 10.1038/srep36850 (2016).

**Publisher’s note:** Springer Nature remains neutral with regard to jurisdictional claims in published maps and institutional affiliations.

## Supplementary Material

Supplementary Information
