# Supplementary material for: Downregulation of Aedes aegypti chromodomain helicase DNA binding protein 7/Kismet by Wolbachia and its effect on dengue virus replication
Source: Sci Rep. 2016 Nov 9;6:36850. doi: 10.1038/srep36850 (PMC5101808; doi:10.1038/srep36850)

Downregulation of *Aedes aegypti* chromodomain helicase DNA binding protein 7/Kismet by *Wolbachia* and its effect on dengue virus replication

Sultan Asad, Sonja Hall-Mendelin and Sassan Asgari

Fig. S1 Diagramed illustration of AeCHD7 conserved domains: Conserved domains were found in AeCHD7 by putting AAEL002230-PA amino acid sequences as query in NCBI conserved domain finder, and the domains characteristic of CHD7 proteins are shown. Red color shows chromodomain, green color shows SNF2 domain and yellow color shows BRK domain.

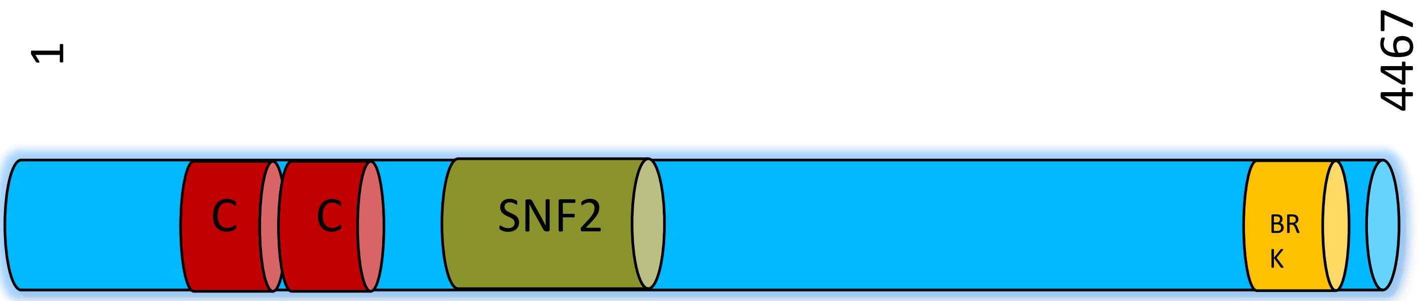

Conserved Domains

Chromodomains (Red):	1781-1821; 1843-1885
SNF2 (Green):	1933-2220
BRK (Yellow):	4137-4174

Fig. S2: A) Table of protein sequences used in this study. **B)** Maximum likelihood phylogenetic tree computed for the protein sequences in (A).

A

Species Name	Uniprot ID	Identity
Aedes aegypti	Q17IV5	100%
Culex quinquefasciatus	B0W8T1	74%
Drosophila melanogaster	Q9VPL9	59%
Homo sapiens	Q9P2D1	54%

B

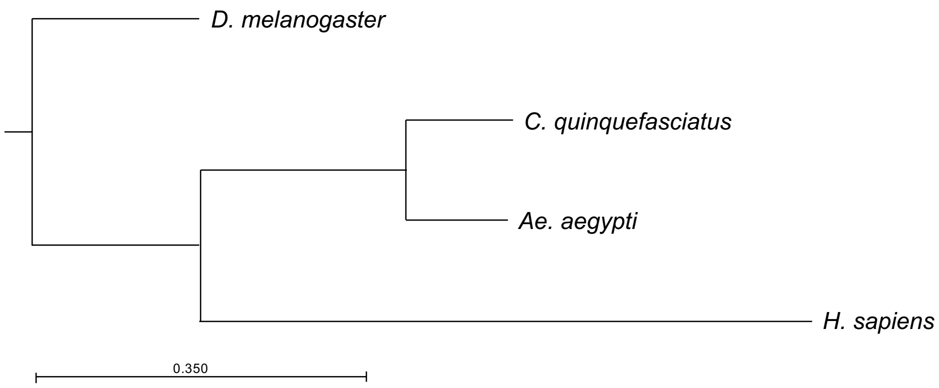

Supplement: Supplementary Information [file srep36850-s1.pdf]
